# Sectional Fixed Orthodontic Extrusion Technique in Management of Teeth with Complicated Crown-Root Fractures: Report of Two Cases

**DOI:** 10.1155/2018/8715647

**Published:** 2018-01-18

**Authors:** S. Nagarajan M. P. Sockalingam, Katherine Kong Loh Seu, Halimah Mohamed Noor, Ahmad Shuhud Irfani Zakaria

**Affiliations:** Department of Operative Dentistry, Faculty of Dentistry, National University Malaysia (UKM), Jalan Raja Muda Abdul Aziz, 50300 Kuala Lumpur, Malaysia

## Abstract

Complicated crown-root fractures account for a small percentage of traumatic dental injuries seen in children; however, management of these injuries can be very challenging to clinicians. Factors such as complexity of the injury, patient's age and dentition stage, patient's cooperation, and parental demands may have some bearing on the type of treatment undertaken and its outcomes. In some children, these injuries may have significant impact on their quality of life. The purpose of this article is to describe two cases of complicated crown-root fracture which were successfully managed through orthodontic extrusion using a sectional fixed orthodontic technique. The basis for the treatment technique and its favourable outcomes were highlighted with its advantages and drawbacks.

## 1. Introduction

Dentoalveolar trauma accounts for 76% of cases seen among children with maxillofacial injuries [1]. In the context of dental injuries alone, the prevalence has been reported to be between 15 and 20% in the permanent dentition of children and adolescents [2, 3]. A large proportion of the dental injuries was due to accidental fall and sport-related activities [4].

Of the different types of dental injuries, the crown fracture which accounts for a third of the injuries is the commonest type reported. In contrast, crown-root fracture only represents 0.3–5% of the injuries stated [5]. Crown-root fracture involves the fracture of enamel, dentin, and cementum with or without the involvement of the pulp [6]. Both the upper central and lateral incisors are the commonest teeth affected by dental injuries [3, 7].

Crown-root fracture especially the complicated type with pulp involvement is one of the most difficult types of dental injury to treat. Factors such as the complexity and direction of fracture, size and mobility of the fractured tooth fragment, subgingival extension of the fracture line, stage root development, alveolar fracture, soft tissue injuries, and the pulp status of the affected tooth at the time of presentation may contribute toward the outcomes of the treatment [8]. Besides these tooth-related factors, additional considerations such parental demand and attitude, patient's cooperation and medical condition, oral condition, and teeth alignment may dictate the possible treatment options to be considered.

This case report highlighted two clinical cases of complicated crown-root fracture of permanent central incisors in growing children with complex fracture lines. The challenges and possible treatment options with the basis of treatment selection were discussed. In addition, the preparation of the affected teeth prior to the root canal treatment and the use of sectional fixed orthodontic extrusion technique were also explained.

## 2. Case Report 1

An eight-year-old girl was referred by her general dental practitioner (GDP) to the National University of Malaysia (UKM) Paediatric Dental Clinic for management of an upper anterior tooth with a complicated crown-root fracture. The tooth fractured two days earlier due to a fall at a poolside. The patient was medical fit and healthy.

**Figure 1:**
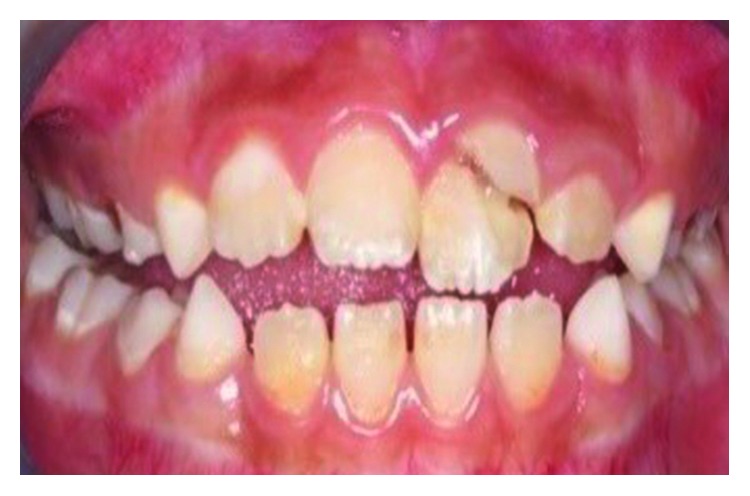
Pretreatment photograph of tooth 21 with crown-root fracture (frontal view).

**Figure 2:**
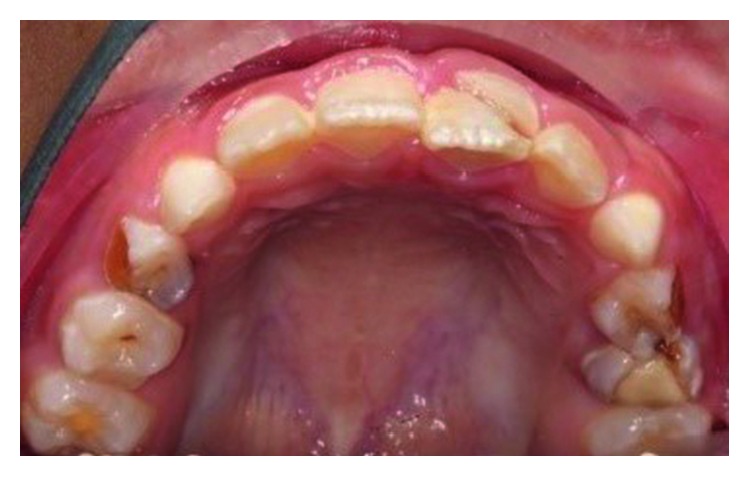
Pretreatment photograph of tooth 21 with crown-root fracture (palatal view).

**Figure 3:**
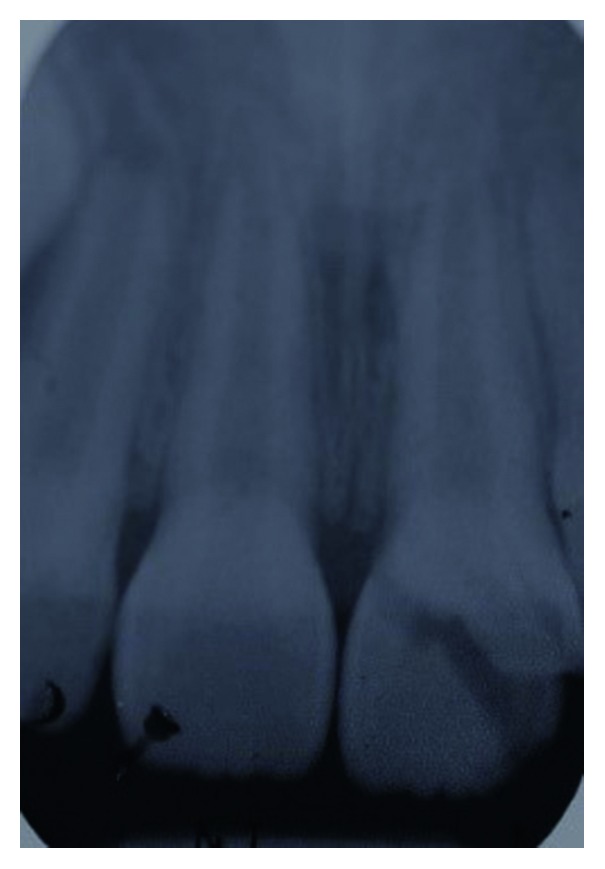
Periapical radiograph of tooth 21 showing the extent of the crown-root fracture.

Clinical examination revealed an oblique fracture from the labial surface of the upper left permanent central incisor (tooth 21) extending palatally 3.5 mm beneath the gingival margin (Figures 1 and 2). The upper coronal two-thirds of the crown structure was mobile, and the tooth did not response to sensibility testing. A periapical radiograph showed evidence of a crown-root fracture of the tooth 21, and the tooth has an immature apex (Figure 3).

**Figure 4:**
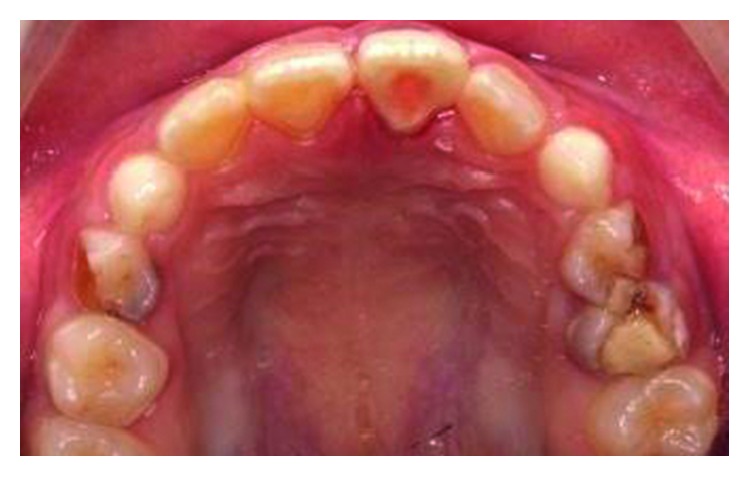
Location of the root canal treatment access on the palatal of the fractured tooth 21 after repositioning of the fractured fragments.

**Figure 5:**
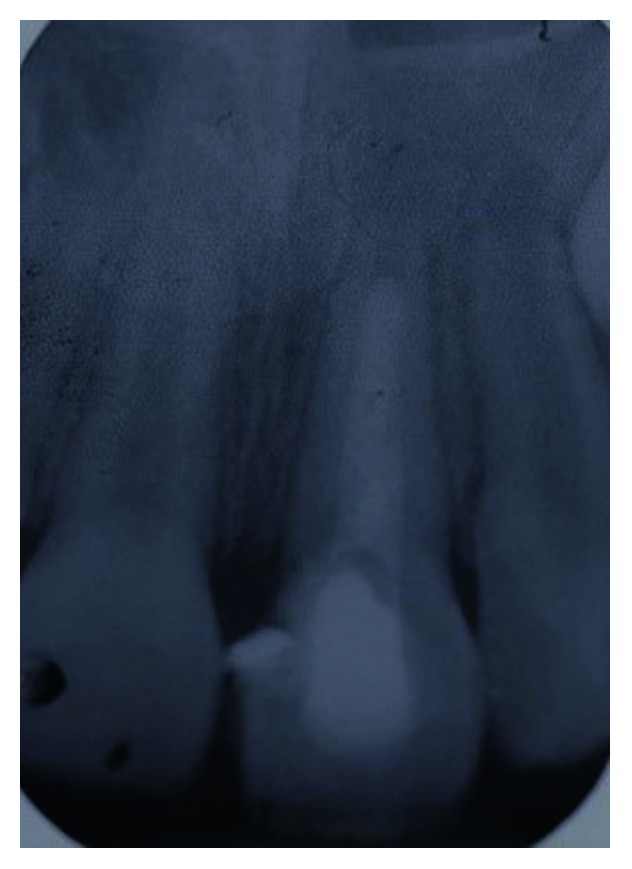
Root canal of tooth 21 filled with nonsetting calcium hydroxide.

**Figure 6:**
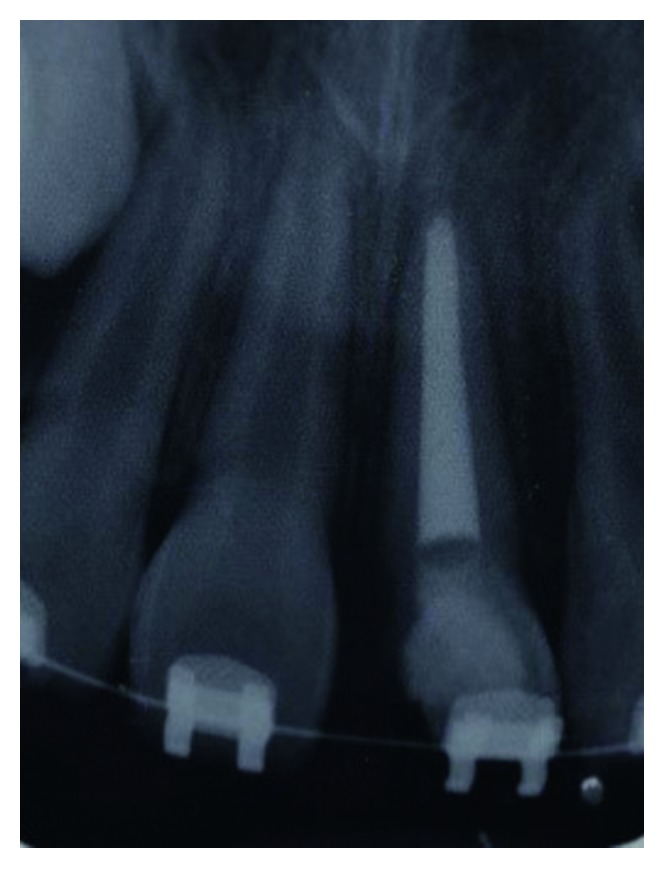
Obturation of the root canal of tooth 21 with bioceramics and gutta-percha.

The fractured crown was pushed into its original position with gentle pressure with digits and held in position by composite resin during the initial stages to allow commencement of the root canal treatment. This allowed minimal interference of the palatal gum tissue and therefore reduced the risk of contamination. An access cavity was made through the crown (Figure 4). The canal was chemomechanically prepared, dried, and filled with nonsetting calcium hydroxide (Figure 5). A week later, apexification was carried out, where the canal was filled with 4 mm of a bioceramic material (EndoSequence®, BC RRM Fast Set Putty^TM^, Brasseler, USA) plug. The following week, the canal was obturated with thermoplasticised GP, and the access cavity was restored with the self-cured GIC (Figure 6).

**Figure 7:**
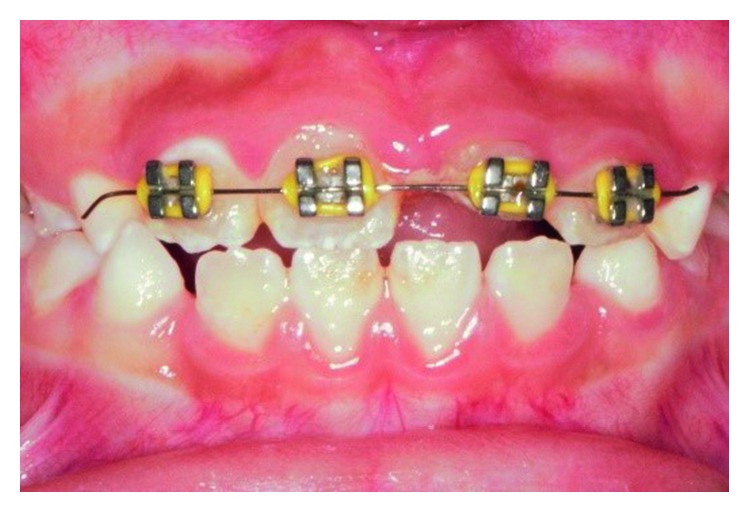
Sectional fixed orthodontic appliance in place for extrusion of fractured tooth 21.

**Figure 8:**
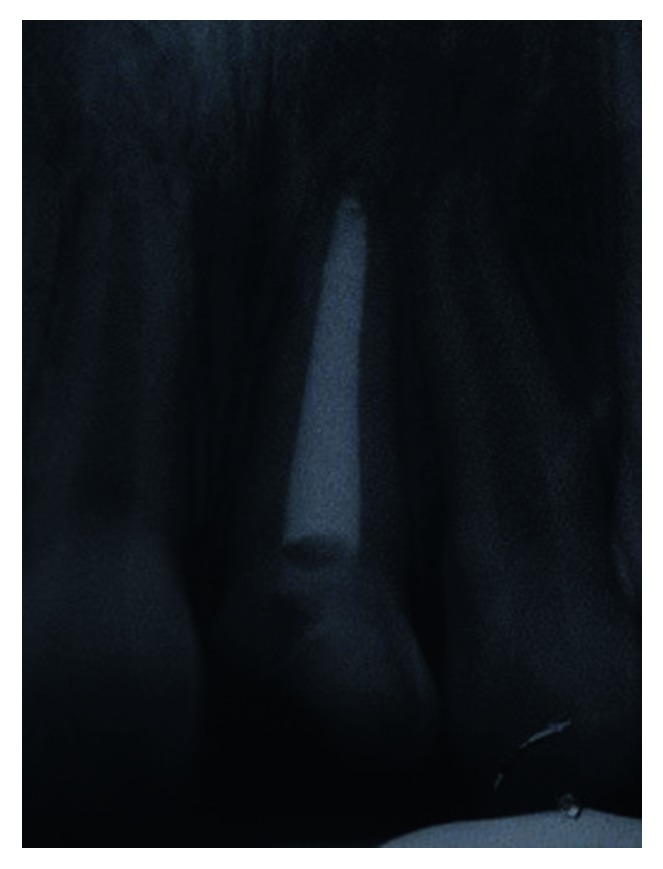
Periapical radiograph of tooth 21 following extrusion prior to reattachment of the fractured fragment of the tooth.

**Figure 9:**
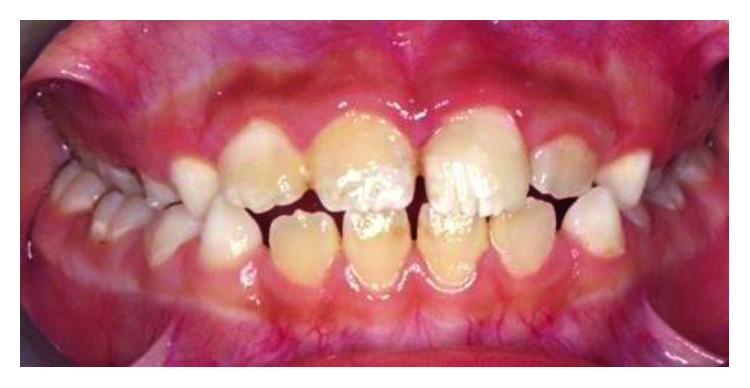
Final restoration of tooth 21 using the patient's own fractured fragment of the tooth.

Subsequently, in order to allow adequate exertion of the orthodontic forces to the root portion, the coronal crown fragment had to be removed and kept in a container of normal saline for hydration. The missing palatal aspect of the crown was filled with self-cured glass ionomer cement from the base of the fracture to above the gingival margin to prevent gingival ingrowth. Following that, four brackets were placed on the labial surfaces of the upper incisors. Extrusion of the fractured tooth was initiated with a sectional fixed orthodontic technique using a 0.014 × 0.025 rectangular nickel-titanium (NiTi) archwire (Figure 7). The patient was reviewed every month. After 4 months, the palatal fracture margin of the tooth was raised to the gum level. At this stage, the brackets were removed and a periapical radiograph was taken to assess the root filling and the root status. The periapical radiograph did not show any remarkable changes (Figure 8). The stored coronal fragment was reattached to the extruded tooth with composite resin (Figure 9). A supracrestal fibrotomy was performed around the extruded tooth with a surgical scalpel blade, and the tooth was splinted on its palatal aspect with the adjacent teeth using a composite-wire splint for 6 months. Post-op reviews at 3, 6, and 12 months of the tooth did not reveal any unremarkable clinical changes.

## 3. Case Report 2

A ten-year-old was brought to the to the National University of Malaysia (UKM) Paediatric Dental Clinic a month after an alleged fall at school by his mother. The patient had some discomfort during eating and felt there is something loose behind his front tooth. He was medically fit and well.

**Figure 10:**
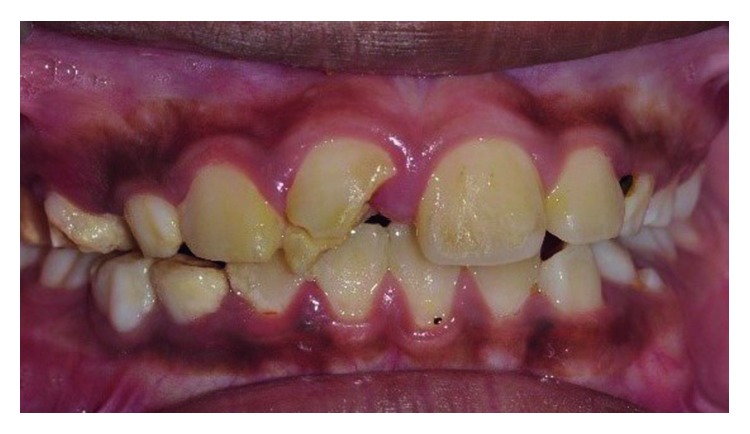
Pretreatment photograph of tooth 11 with crown-root fracture (frontal view).

**Figure 11:**
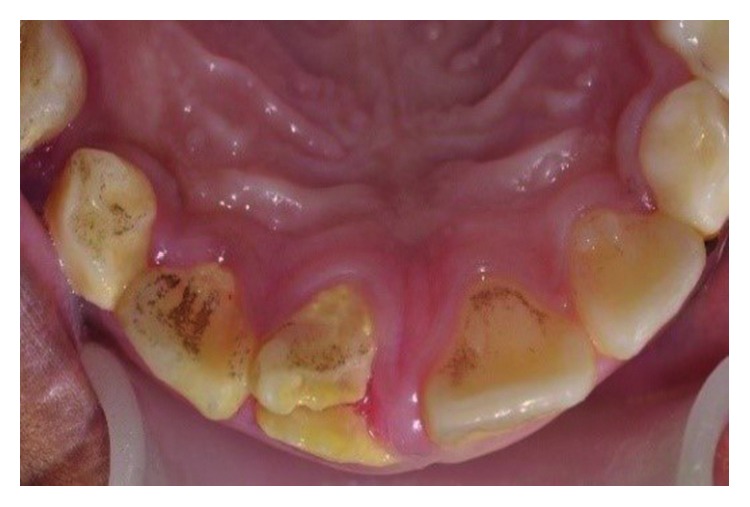
Pretreatment photograph of tooth 11 with crown-root fracture (palatal view).

**Figure 12:**
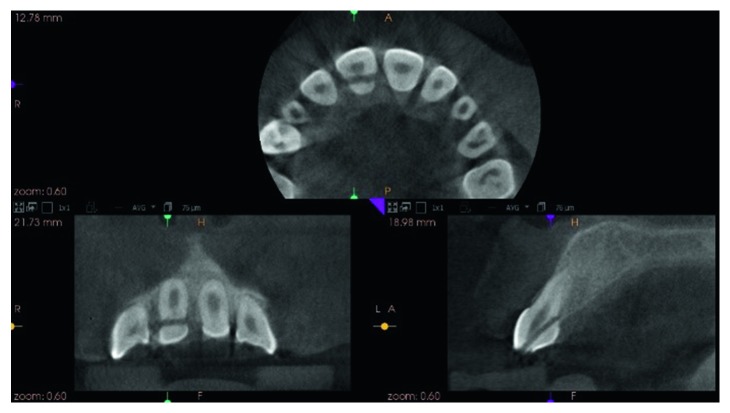
Cone beam computed tomographic view of tooth 11 in various planes showing the extent of the fracture.

Clinical examination showed a crown-root fracture of the upper right permanent central incisor (tooth 11) (Figure 10). The oblique fracture line extended in a labiopalatal direction with the palatal margin extending 3.5 mm subgingivally (Figure 11). Cone Beam Computer Tomography (CBCT) images of the tooth showed a complicated crown-root fracture, and the tooth has an almost matured root apex (Figure 12). Sensibility test was negative.

**Figure 13:**
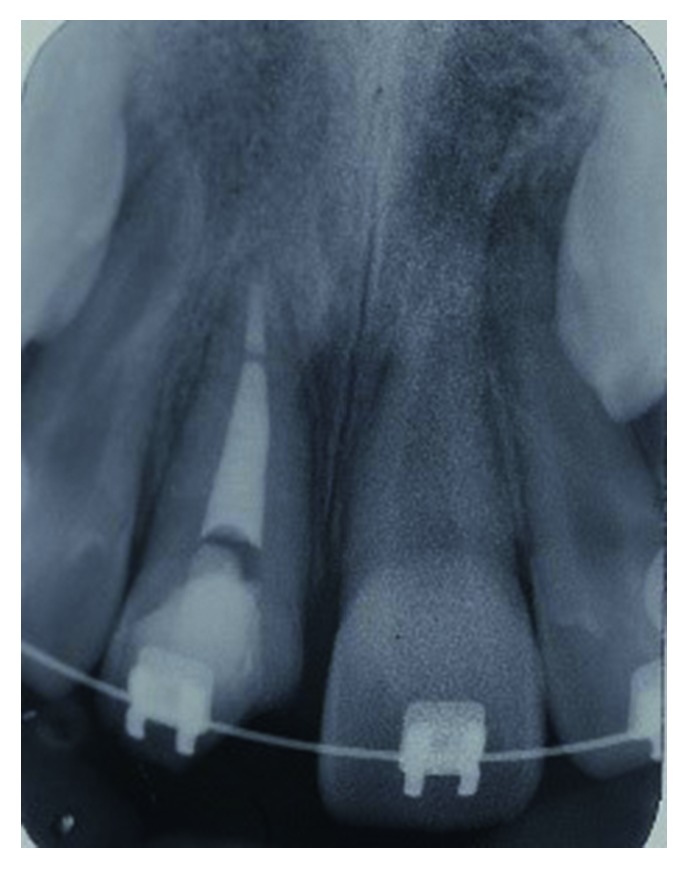
Obturation of the root canal of tooth 11 with bioceramics and gutta-percha.

**Figure 14:**
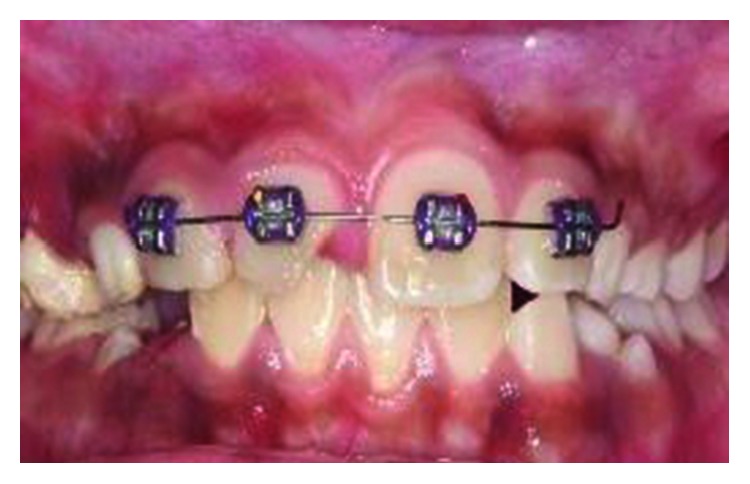
Sectional fixed orthodontic appliance in place for extrusion of tooth 11.

**Figure 15:**
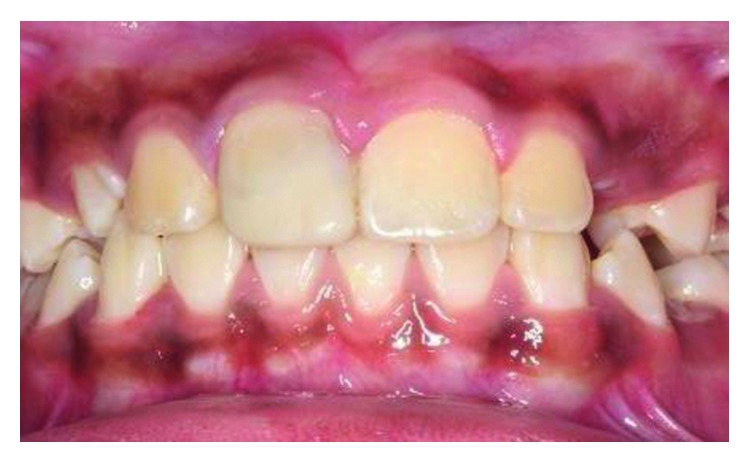
Composite restoration of the fractured tooth 11 after orthodontic extrusion.

**Figure 16:**
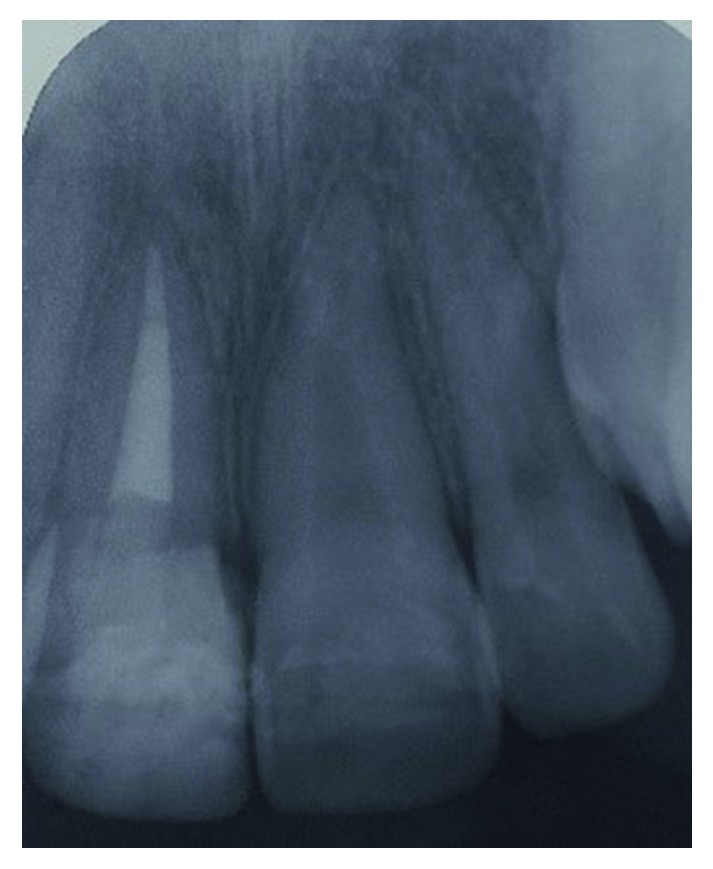
Postoperative periapical radiograph of the fractured tooth 11 at 12 months after extrusion.

The palatal mobile tooth fragment was removed under local anaesthesia, and palatal fracture margin was identified. After haemostasis control, self-cured GIC was placed on the palatal aspect of the tooth similar to that in Case 1. A week later, a root canal treatment was initiated with chemomechanically preparation. Thereafter, the canal was filled with 4 mm of a bioceramic material (EndoSequence®, BC RRM Fast Set Putty^TM^, Brasseler, USA) plug. The following week, the canal was obturated with thermoplasticised GP, and four orthodontic brackets were placed on the labial surfaces of tooth 12 to tooth 22 (Figure 13). A short span 0.014 × 0.025 rectangular nickel-titanium (NiTi) archwire was used to extrude the tooth fractured palatal margin further gingivally (Figure 14). The patient was reviewed monthly. Five months later, sufficient amount of the palatal margin of the fractured tooth was clinically visible for composite build-up (Figure 15). A palatal wire-composite splint was placed for 6 months following a supracrestal fibrotomy around the tooth. The tooth was successfully reviewed at 3-, 6- (Figure 16), and 12-month intervals without any evidence of pathosis.

## 4. Discussion

Two important questions that are often thought of by clinicians pertaining dental injuries are, Is it possible to save the injured tooth? Is it desirable to save the injured tooth? A multitude of problems such tooth, patient, and parental related factors may dictate the decision process to either save the tooth or extract it [8]. Another concern in a growing child, losing a tooth at such a tender age may have a significant effect on the child's quality of life. Studies have shown that traumatic dental injuries can bring about a strong and prolong impact on the emotion and social well-being on the affected children [9, 10]. Parents also often hope in desperation that clinicians can do something to salvage the traumatised tooth.

There are few treatment options available to manage teeth with complicated crown-root fractures [11, 12]:

- Removal of the fractured coronal fragment and restoration of the tooth if the fracture line has not encroached into the biologic width.
- Removal of the coronal fragment and supplement with gingivectomy or/and osteotomy to expose the fracture line in order to establish biologic width prior to restoration.
- Removal of the coronal fragment and initiation of endodontic treatment and restoration of tooth with a postcrown.
- Removal of the coronal fragment and initiation of endodontic treatment and followed by either orthodontic or surgical extrusion of the apical fragment prior to restoration with a postcrown.
- In severe crown-root fracture, the tooth may have to be extracted and replaced with a removal or fixed prosthesis.

If a decision to save the tooth is taken, one should ensure the restorability of the remaining tooth structure after the removal of the mobile coronal fragment and availability of adequate root length. Generally, teeth with crown-root fractures require a multidisciplinary intervention especially if the teeth need to be saved. Three main issues need to be considered prior to treatment:

- If the tooth needs RCT, how to prevent contamination of the canal form the subgingival tissue?
- How to bring the subgingival fracture margin to equigingival or supragingival level?
- How to provide a lasting and aesthetic restoration that not only provides good coronal seal but also has self-cleansing margins?

In the cases presented, both the teeth had complicated crown-root fracture and required RCT. One of the main challenges in carrying out RCT on crown-root fractured teeth is bleeding and crevicular fluid contamination from the gums which usually occurs after removal of the mobile coronal fragment. Two different approaches were undertaken to minimise the degree of contamination: in Case 1, the fractured crown was repositioned and held together in the reduced position with composite resin. The RCT was carried through the fractured crown. In Case 2, the coronal fragment was too loose for reattachment and had to be removed. GIC was placed on the palatal defect up to the supragingival level after control of gum bleeding. The GIC forms a continues rim with the tooth and allowed the gums to heal without any ingrowth into the defective area prior to the commencement of the RCT. Conventional GIC was used because it has better biocompatibility to gingivae than resin-modified GIC [13]. Since both the traumatised teeth had near completion of the root apices and large pulp canals, apexification was carried out. In both cases, apexification was carried out with a bioceramic material (EndoSequence®, BC RRM Fast Set Putty^TM^, Brasseler, USA) after chemomechanical debridement of the canals. The Endo Sequence Root Repair Material is a calcium silicate-based cement which exhibits high biocompatibility and has antibacterial and osteogenic properties. It can be used as an alternative to mineral trioxide aggregate [14, 15].

Another treatment issue that required much attention was the subgingival fracture margins. In order to provide good and self-cleansing restoration, the restorative margin should be either equigingival or supragingival. As both the traumatic teeth had fractures extending subgingivally and had encroached into the biologic width, extrusion of the apical portion of the teeth was decided to bring the fractured margins close to the desired gum level. In order to achieve this, two options were considered, either the use of orthodontic extrusion or surgical extrusion. Although both methods have proven clinical successes to extrude teeth with minimal complications [16–18], orthodontic extrusion with light forces was used in the current cases due to the patients' age. A sectional fixed orthodontic technique was used for extrusion with four brackets and a short NiTi (0.014 × 0.025) rectangular wire. Use of this technique offers many advantages such good patient compliance, easy access, and less number of teeth involved in bracket placement, easy cleaning, and able to deliver the desired result. Nevertheless, orthodontic extrusion is a much slower process than surgical extrusion, and supracrestal fibrotomy with a retention period of 6 months may be necessary to allow periodontal fibres reattachment and healing [19]. Otherwise, relapse can happen over time.

With regard to restoration of teeth with crown-root facture, factors such as the extent of the fracture, availability of tooth structure, presence of the tooth fragment, occlusion, aesthetic, and patient's age and cooperation may dictate the type of restoration needed. If the fractured coronal fragment is large and available, it could be reused to restore the tooth as demonstrated in Case 1. Another option is to do a composite build-up or composite crown with or without a postcore. Often in a growing child, a ceramic extracoronal crown is not considered as the restorative margins become visible with growth. On the other hand, composite-based restorations allow easy repair and adjustment.

## 5. Conclusion

The current cases demonstrated the application of multidisciplinary approach in the management of teeth with crown-root fractures. The management displayed three main areas of expertise: the endodontics, orthodontics, and restorative. Each tooth with complicated crown-root fracture has its uniqueness and challenges that need to be taken into consideration during the treatment planning stage. The sectional fixed orthodontics used produced a favourable extrusion of the crown-root fractured teeth and may offer a better alternative to be used than other forms of modified removal appliances.
